# Detection and analysis of drug–drug interactions among hospitalized cardiac patients in the Mohammed V Military Teaching Hospital in Morocco

**DOI:** 10.11604/pamj.2018.29.225.14169

**Published:** 2018-04-24

**Authors:** Hicham Fettah, Youssef Moutaouakkil, Mohamed Reda Sefrioui, Badreddine Moukafih, Yassir Bousliman, Ahmed Bennana, Jamal Lamsaouri, Sanaa Makram, Yahia Cherrah

**Affiliations:** 1 Laboratory of Pharmacology, Toxicology, Faculty of Medicine and Pharmacy, Rabat Institute, University Mohamed V, Rabat-Morocco; 2 Laboratory of Medicinal Chemistry, Faculty of Medicine and Pharmacy, Rabat Institute, University Mohamed V, Rabat, Morocco

**Keywords:** Cardiology, drug–drug interactions, prevalence, theriaque

## Abstract

**Introduction:**

Drug-drug interactions (DDIs) are defined as two or more drugs interacting in such a manner that the effectiveness or toxicity of one or more drugs is altered. Patients with cardiovascular disorders are at higher risk for DDIs because of the types and number of drugs they receive. The aim of the present study was to assess the prevalence of DDIs in patients admitted to the cardiology department of a hospital in Morocco.

**Methods:**

A prospective observational study from June 2016 to September 2016 was carried out in the cardiology department of a hospital in Morocco. Those patients who were taking at least two drugs and had a hospital stay of at least 48 hours were included in the study. The medications of the patients were analysed for possible interactions. All the prescriptions of the study population were screened for drug-drug interactions using a computerized DDI database system (Theriaque^®^).

**Results:**

During the study period, 138 patients were included; 360 interactions were detected among 94 patients, with an average number of drugs taken of 5.2. The prevalence of DDIs was estimated at 68.11%, the most common of which concerned Kardegic/Plavix (12.22%), Kardegic/Heparin (8.33%), and Lasilix/Spironolactone (5.83%). Among the 726 prescribed drugs, (372 [51.24%]) were drugs of the cardiovascular system, followed by blood and hematopoietic organ drugs (288 [39.67%]) according to the Anatomical Therapeutic Chemical Classification codes. These interactions were categorized on the basis of level of severity: interactions with major severity accounted for 11.11% (40) of the total DDIs while those with moderate and minor severity accounted for 37.22% (134) and 51.66% (186), respectively.

**Conclusion:**

This study reports the prevalence of DDIs in patients admitted to the cardiology department of a hospital in Morocco. This study shows that DDIs are frequent among hospitalized cardiac patients and highlights the need to screen prescriptions of cardiovascular patients for possible DDIs, as this helps in their detection and prevention.

Pan African Medical Journal – ISSN: 1937- 8688 (www.panafrican-med-journal.com)

Published in partnership with the African Field Epidemiology Network (AFENET). (www.afenet.net)

Pan African Medical Journal – ISSN: 1937- 8688 (www.panafrican-med-journal.com)

Published in partnership with the African Field Epidemiology Network (AFENET). (www.afenet.net)

## Introduction

Drug-drug interactions (DDIs) are defined as two or more drugs interacting in such a manner that the effectiveness or toxicity of one or more drugs is altered [1]. In every DDI, we should distinguish between the molecule which is at the origin of an interaction (that is to say modifying the other drugs' concentration and action) and the molecule whose altered concentration, resulting from the same interaction, ultimately leads to the desired or undesired effects (in the case of a narrow therapeutic index drug) [2]. Various studies have shown that adverse drug reactions are responsible of 0.5% to 2% of consultations in ambulatory medicine and are involved in 4% to 10% of hospitalizations [3, 4]. It has been estimated that 1/3 of the severe adverse effects are due to negligence and thus can be preventable; 20% of these side effects are caused by DDIs [4]. The prevalence of cardiovascular diseases has increased in the last decade and they are considered as the primary cause of mortality in the world [5]. Indeed, cardiac tropism drugs are the most common cause of drug-related iatrogenia, accounting for 12 to 28% of cases [6] and are the most implicated drugs in DDIs. Some 1500 out of 300 000 documented DDIs are considered clinically significant, the most serious of which appear in the field of cardiology [7]. Drug-related iatrogenia in their totality are the subject of various studies because of their impact in terms of health economics, but the number of specific studies on DDIs is limited. Although drug interactions are reported to be common in cardiology, there is no published report of the prevalence of such interactions among Moroccan cardiac patients. The aim of the present study was to assess the prevalence of DDIs in patients admitted to the cardiology department of a hospital in Morocco.

## Methods

A prospective observational study was carried out for a period of four months (between June and September 2016) at the Mohammed V Military Teaching Hospital in Morocco. Patients who were admitted consecutively to the cardiology department were included in the study. Only patients with two or more drugs prescribed during the hospitalization were selected for the study. The studied population comprised all patients aged 20 years or older admitted to the hospital with a length of stay greater than 48 hours. Demographic information (age and gender), number of drugs taken and length of hospital stay were obtained from the clinical records. All the prescriptions of the study population were screened for drug-drug interactions using a computerized DDI database system (Theriaque^®^) [8, 9]. The drugs were classified according to the Anatomical Therapeutic Chemical (ATC) classification codes [10]. The interactions observed were classified into mild, moderate and severe and the data on severity was obtained from the DDI data of the drug database [8, 9]. The results of the study were reported using the Microsoft Excel software.

## Results

**Figure 1:**
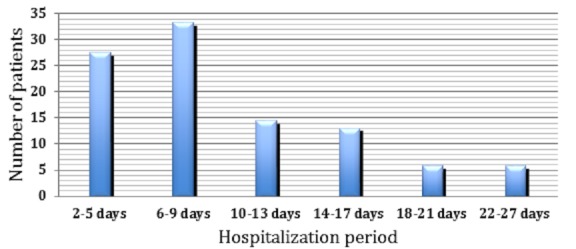
Distribution of patients according to the hospitalization period

**Figure 2:**
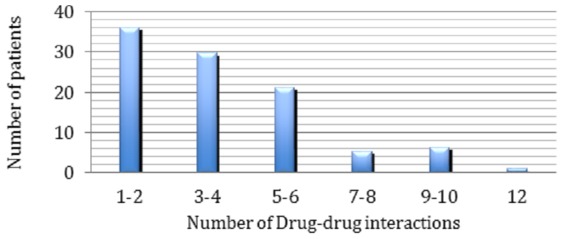
Number of drug-drug interactions by patient

A total of 138 patients were analysed during the study period, with a male predominance of (73.9%) and a sex ratio of 0.35. The median patient age was 61.7 years old, with ages ranging from 20 to 92 years old. The average stay in hospital was 10 days (Figure 1). Most patients had cardiovascular diseases with a predominance of ischemic heart disease (42.75%, n: 59) followed by hypertension (39.85%, n: 55) and atypical chest pain (5.79%, n: 8) (Table 1). The analysis of prescriptions allowed us to identify 360 interactions among 94 patients, with an average number of drugs taken of 5.2 (Figure 2). The prevalence of DDIs was estimated at 68.11%, the most common of which concerned Kardegic/Plavix (12.22%), Kardegic/Heparin (8.33%), and Lasilix/Spironolactone (5.83%). Among the 726 prescribed drugs, 372 (51.24%) were drugs of the cardiovascular system, followed by blood and hematopoietic organ drugs (288 [39.67%]) according to the ATC classification codes (Table 2). These interactions were categorized on the basis of level of severity: Interactions of major severity accounted for 11.11% (40) of the total DDIs while those of moderate and minor severity accounted for 37.22% (134) and 51.66% (186), respectively. The clinical consequences of these drug interactions in most cases were hypotension, hypoglycaemia, increased risk of bleeding, and renal dysfunction. The classifications of the potential DDIs were made based on their pharmacodynamic or pharmacokinetic mechanisms. Among 360 DDIs, 77.78% were pharmacokinetic and 22.22% were pharmacodynamic.

**Table 1:** Diagnosis of patients with cardiovascular diseases in Mohammed V Military Teaching Hospital in Morocco (n=138)

Diagnosis	Number (%)
Ischemic heart disease	59 (42.75)
Hypertension	55 (39.85)
Atypical Chest Pain	8 (5.79)
Unstable Angina	7 (5.07)
Atrial Fibrillation	4 (2.89)
Coronary heart disease	3 (2.17)
Congestive heart failure	2 (1.44)

**Table 2:** The anatomical therapeutic chemical classification of prescribed drugs

The anatomical therapeutic chemical classification of drugs	Drug	Number	Percentage (%)
Cardiovascular system = 51.24 %	Ramipril	82	11.29
	Spironolactone	63	8.68
	Lasilix	56	7.71
	Carvedilol	28	3.86
	Atenolol	18	2.48
	Molsidomine	18	2.48
	Amlodipine	17	2.34
	Simvastatine	13	1.79
	Perindopril	12	1.65
	Valsartan	10	1.38
	Cordarone	8	1.1
	Propranolol	7	0.96
	Risordan	6	0.83
	Acebutalol	5	0.69
	Ivabradine	4	0.55
	Natispray	4	0.55
	Amiloride/ | Hydrochlorothiazide	3	0.41
	Bisoprolol	3	0.41
	Captopril	3	0.41
	Digoxine	3	0.41
	Irbesartan	3	0.41
	Flecaine	2	0.28
	Indapamide	1	0.14
	Losartan	1	0.14
	Nebivolol	1	0.14
	Rosuvastatine	1	0.14
blood and blood forming organs = 39.67%	Kardegic	103	14.19
	Heparin	75	10.33
	Plavix	63	8.68
	Sintrom	47	6.47
Digestive tract and metabolism = 5.79%	Insuline	25	3.44
	Eucarbon	5	0.69
	Gliclazide	3	0.41
	Glucophage	3	0.41
	Diffu-K	2	0.28
	Smecta	2	0.28
	No-spa	1	0.14
	prednisolone	1	0.14
Muscle and skeleton = 1.24%	Naproxen	5	0.69
	Allopurinol	3	0.41
	Colchicine	1	0.14
Nervous system = 0.96%	Laroxyl	4	0.55
	Deroxat	1	0.14
	Modopar	1	0.14
	Piribedil	1	0.14
Genitourinary system and sexual hormones = 0.41%	Alfuzosine	3	0.41
Anti-infection drugs for systemic use = 0.28%	Ciprofloxacine	1	0.14
	Flucloxacilline	1	0.14
Dermatological drugs = 0.28%	Hydrocortisone	2	0.28
Antiparasitics, Insecticides = 0.14%	Nivaquine	1	0.14

## Discussion

This study revealed the overall prevalence of clinically important DDIs in the cardiology department to be 68.11%. The value obtained in the present study is higher compared with the study by Patel et al in India, who reported an incidence rate of 30.23% [11]. These differences might be because our study took into consideration all drug interactions of moderate and minor severity in contrast to the other studies. In our study, there were 726 drugs prescribed to 138 patients. The average number of drugs per patient was 5.2. This can be explained by the simultaneous use of many drugs. The frequent addition of new drugs makes these patients vulnerable to DDIs. The most common interacting pairs identified were Kardegic/Plavix (12.22%), Kardegic/Heparin (8.33%), and Lasilix/Spironolactone (5.83%). The concurrent use of Kardegic with these potentially interacting medications necessitates the tailoring of doses to the individual patient and careful monitoring by the patient and health care provider for signs of bleeding. The classes of drugs most commonly involved in DDIs, according to the ATC classification, were cardiovascular system and blood and blood-forming organs. These two classes together accounted for 90.91% of the identified DDIs. This might be due to the frequent use of these drug classes among the cardiac patients in the present study. The classifications of the potential DDIs were made based on their pharmacodynamic or pharmacokinetic mechanisms. Among 360 DDIs, (77.78%) were pharmacokinetic and (22.22%) were pharmacodynamic. This figure correlates with the results of similar studies [12-14]. In our study, the significant association of DDIs with old age, number of drugs taken, longer hospital stay, and multiple disease states is in accordance with published reports [15,16]. These findings suggest that patients with these risk factors are more exposed to DDIs. We recommend that such patients be monitored closely in order to avoid the grave consequences of DDIs. Of the total DDIs identified, the majority were of minor or moderate severity; these findings are different from the reports of spontaneous reporting studies [17, 18]. Ischemic heart diseases are a major cause of hospitalization. This can be due to the number of co-morbidities or to the high frequency of these diseases in the Moroccan population. This predominance of heart disease may explain the increase in the length of stay at the cardiology department [19]. The suggested action to be taken in most cases was to strictly control blood glucose; monitor renal function, serum potassium level and coagulation parameters; and watch for clinical signs of hypoglycaemia and bleeding.

## Conclusion

This study reports the prevalence of DDIs in patients admitted to the cardiology department of a hospital in Morocco. The study also examined patient and drug characteristics, and the severity of DDIs. This study shows that DDIs are frequent among hospitalized cardiac patients and highlights the need to screen prescriptions of cardiovascular patients for DDIs, as this helps in the detection and prevention of possible adverse drug interactions.

### What is known about this topic

- The drug-drug interaction increases as the number of concomitant medications increases;
- Patients with cardiovascular diseases are at higher risk for drug-drug interactions because of the types and number of drugs they receive.

### What this study adds

- The present study has recorded a high prevalence of DDIs in cardiology department;
- The classes of drugs most commonly involved in DDIs were cardiovascular system and blood and blood-forming organs;
- Most of the interactions were of minor or moderate severity.

## Competing interests

The authors declare no competing interests.
